# Microstructure and Processing Performance of Brazed Diamond Micro-Powder Grinding Wheel with Ni-Based Filler Alloy

**DOI:** 10.3390/ma19091800

**Published:** 2026-04-28

**Authors:** Shuyi Wang, Haozhong Xiao, Bing Xiao

**Affiliations:** 1 School of Naval Architecture & Intelligent Manufacturing, Jiangsu Maritime Institute, Nanjing 211170, China; 20231136@jmi.edu.cn; 2 College of Mechanical and Electrical Engineering, Nanjing University of Aeronautics and Astronautics, Nanjing 210016, China; meebxiao@nuaa.edu.cn

**Keywords:** diamond micro-powder, cemented carbide, interface microstructure, grinding performance, surface roughness

## Abstract

In this study, a brazed diamond micro-powder grinding wheel with Ni-based filler metal was fabricated, which achieved one-step grinding forming of YG-6 cemented carbide rods. The interfacial microstructure, elemental diffusion behavior, and interfacial phases of the brazed diamond micro-powder joint were systematically characterized. Furthermore, the machining performance of the brazed diamond micro-powder grinding wheel was comprehensively evaluated in combination with its service life and the surface roughness of the machined YG-6 cemented carbide rods. The results show that the Ni-based filler exhibits good wettability to diamond micro-powder particles, and the diamonds have a reasonable protrusion height in the filler layer, with no graphitization observed on the surface of the brazed diamonds. During the brazing process, the active element Cr continuously segregates toward the diamond surfaces and reacts progressively with dissolved C atoms on the diamond surfaces, eventually forming a lath-shaped Cr–C compound layer on the diamond surfaces. XRD results identify this compound as Cr_3_C_2_. Elemental diffusion occurs between the filler layer and the steel substrate, forming a Fe–Ni solid solution diffusion zone. Consequently, the Ni-based filler forms a reliable chemical metallurgical bond with both the diamond micro-powder particles and the steel substrate. The as-prepared brazed diamond micro-powder grinding wheel exhibits excellent service life: a single wheel can grind more than 1300 YG-6 cemented carbide rods on average before failure. The surface roughness (Ra) of the machined YG-6 cemented carbide workpieces remains below 1.6 μm throughout all processing stages, which satisfies the requirements for one-step precision grinding.

## 1. Introduction

Cemented carbides exhibit excellent material properties, including high hardness, high strength, strong chemical stability, and superior wear resistance, and thus they are widely used in cutting tools, molds, and wear-resistant components [1,2]. Among various cemented carbides, WC-Co cemented carbide is the preferred material for the fabrication of cutting tools due to its outstanding cutting performance [3,4]. However, the high hardness and brittleness of WC-Co cemented carbide pose significant challenges to machining; furthermore, its machined surface is prone to cracks and pits, making it a typical difficult-to-machine material [5,6]. Currently, with the continuous emergence of various advanced materials, numerous novel processing technologies have been developed [7,8]. According to existing research and engineering practice, grinding remains the dominant processing method for WC-Co cemented carbide [9,10,11]. This process not only ensures high machining efficiency but also delivers excellent surface quality, meeting the demands of high-precision cutting scenarios. Diamond abrasives are widely used for grinding hard and brittle materials due to their excellent mechanical properties [12,13]. With the development and application of diamond grinding tools, considerable efforts have been devoted to their use in the grinding of cemented carbides [14,15,16]. Traditional diamond abrasive tools mainly include resin-bonded, electroplated, and sintered types. During the grinding of cemented carbides, these tools often suffer from premature dislodgement of diamond abrasives from the tool matrix. This not only reduces machining efficiency but also deteriorates the surface quality of workpieces. Additionally, the low protrusion height of diamond abrasives leads to wheel clogging and consequently increased grinding forces, which adversely affects the improvement of workpiece surface quality and machining accuracy, failing to meet the requirements of precision machining technology for cemented carbides. Brazed diamond abrasive tools use filler alloy as the bonding agent, achieving chemical bonding between diamonds and the matrix at high temperatures [17,18,19,20,21,22]. They offer high bonding strength between diamonds and the matrix, along with high abrasive protrusion height and excellent chip removal. Compared with traditional diamond tools, brazed diamond tools exhibit significantly improved machining performance on brittle and hard materials [23,24].

Diamond micro-powder, as the hardest ultra-fine abrasive, is an ideal choice for grinding brittle and hard materials such as cemented carbides, ceramics, and optical glass. Brazed diamond micro-powder tools combine the high bonding strength of brazing technology with the high-precision machining performance of diamond micro-powder, exhibiting potential for one-step precision grinding of cemented carbides [25,26,27,28]. At present, relevant studies worldwide mainly focus on the brazing mechanism of conventional-sized diamond grains and the fabrication of corresponding brazed diamond tools. However, investigations into the brazing behavior and interfacial characteristics of diamond micro-powder remain insufficient. In particular, research on the one-shot forming machining of cemented carbide using brazed diamond micro-powder tools is even scarcer. The interfacial microstructure of Ni-based brazed diamond micro-powder joints has not been systematically revealed, and systematic studies on the machining performance and workpiece surface quality of such tools during the one-step lapping of cemented carbide are still lacking.

In view of this, 300-mesh diamond micro-powder was used as raw material in this paper to prepare brazed diamond micro-powder tools with Ni-based filler alloy, aiming to achieve one-step high-efficiency grinding of WC-Co cemented carbide with a target surface roughness of Ra ≤ 1.6 μm. The interfacial morphology, elemental diffusion behavior, and surface phase composition of the brazed diamond micro-powder joints were systematically investigated. Additionally, via machining experiments on WC-Co cemented carbide rods, the surface roughness of machined workpieces was measured. Combined with statistics on tool service life, a comprehensive evaluation of the machining performance of the brazed diamond micro-powder tools was conducted.

## 2. Experimental Methodology

### 2.1. Materials and Brazing Process

**Figure 1:**
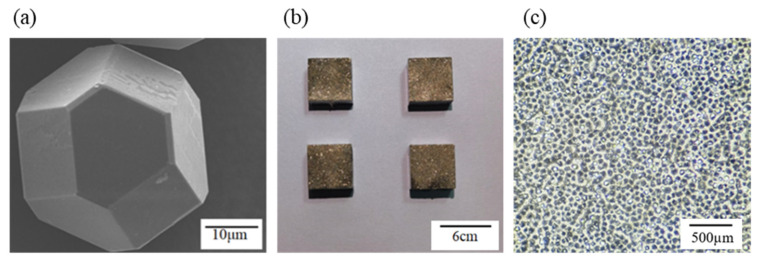
(**a**) Diamond micro-powder, (**b**) as-brazed specimen, (**c**) optical micrograph of brazed diamond micro-powder.

**Figure 2:**
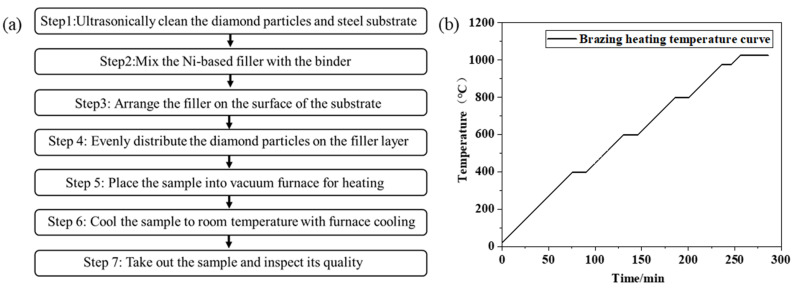
(**a**) Brazing process flow diagram, (**b**) the brazing heating process curve.

Diamond micro-powder with a particle size of 300 mesh (Figure 1a) was adopted, paired with 300/400 mesh BNi-2 alloy powder (composition: Ni–7Cr–4Fe–3Si–3B, wt.%) and 45# steel as the tool substrate. The chemical composition of 45# steel is listed in Table 1. The Ni-based filler alloy mixed with a binder was uniformly applied to the surface of the bulk steel substrate, followed by even spreading of diamond micro-powder onto the brazing layer via a powder spreader. Brazing was conducted in a vacuum furnace (VBF-150, ACX Co., Ltd., Seoul, Republic of Korea) under a vacuum of 10^−2^ Pa, with a heating rate of 5 °C/min. Upon reaching the maximum brazing temperature of 1025 °C, the furnace was held at this temperature for 30 min to ensure sufficient melting of the filler metal. Upon completion of the holding stage, heating was terminated, and the brazed specimens were cooled to room temperature in the furnace before being taken out. The brazing process flow diagram and brazing heating curve are shown in Figure 2. The as-brazed specimen is illustrated in Figure 1b. Optical microscopy observations indicated that the brazed diamond micro-powder was uniformly distributed on the substrate surface without agglomeration (Figure 1c).

### 2.2. Characterization Methods

**Figure 3:**
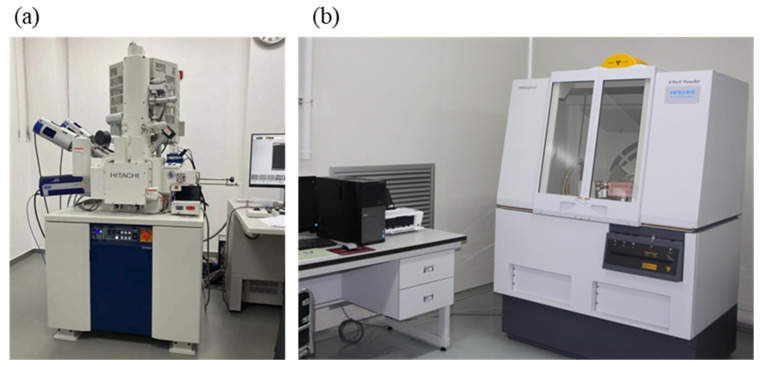
(**a**) Scanning Electron Microscopy, (**b**) X-ray powder diffractometer.

Scanning Electron Microscopy (SEM, Hitachi Regulus 8220, Hitachi High-Tech, Hitachi, Japan) was used to observe the microstructural morphology of brazed diamond micro-powder joints, while Energy-Dispersive Spectroscopy (EDS) was employed to analyze the elemental distribution at the bonding interface. After acid etching with aqua regia of the brazed samples, the brazed diamonds were recovered from the samples, and X-ray diffraction (XRD, Ultima IV, Rigaku Corporation, Akishima, Japan) was utilized to determine the phases of interfacial compounds. The recovered diamond particles were evenly spread on a microscope slide with dimensions of 25 × 35 × 1 mm^3^. For the XRD test, the scanning angle range was set from 30° to 80°, and the scanning speed was maintained at 2°/min. Raman spectroscopy (RS, Horiba HR800, HORIBA, Ltd., Kyoto, Japan, λ = 532 nm) was applied to measure the degree of graphitization of the brazed diamonds. The relevant experimental equipment is presented in Figure 3.

### 2.3. Machining Tests and Surface Roughness Measurement

**Figure 4:**
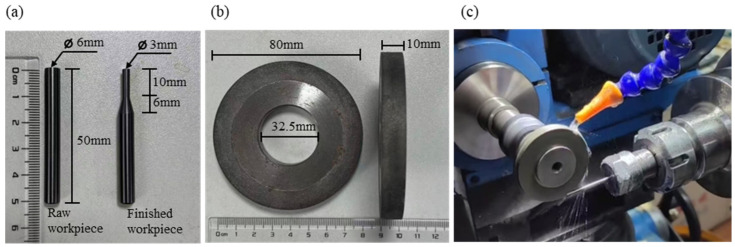
(**a**) Workpiece, (**b**) diamond micro-powder tool, (**c**) machining test.

To evaluate the machining performance of brazed diamond micro-powder grinding wheels with nickel-based brazing filler metal on cemented carbides, YG-6 cemented carbide rods were selected as workpieces, and their mechanical properties are listed in Table 2. The initial morphology of the workpieces is illustrated in Figure 4a, which are cylindrical rods with a length of 50 mm and a diameter of 6 mm. The brazed diamond micro-powder grinding wheels fabricated in this study have the following structural parameters: outer diameter of 80 mm, inner diameter of 33 mm, and end face width of 10 mm, with their morphology shown in Figure 4b. Grinding experiments were performed on a CNC grinding machine (Figure 4c) with the following machining parameters: spindle speed (n) = 3600 r/min, feed rate (vf) = 0.001 m/s, and cutting fluid was applied throughout the grinding process. The morphology of the machined workpieces is presented in Figure 4a: a 10 mm long segment at one end of each workpiece was ground to a diameter of 3 mm, with a 6 mm long inclined step formed. Five sets of grinding wheels (3 wheels per set) were used for the tests. The total number of workpieces machined by each set until wheel failure was recorded to calculate the average service life of each set. After cumulative machining of 1000 workpieces, representative workpieces and grinding wheels were randomly selected. A laser scanning confocal microscope (LSM700, Carl Zeiss MicroImaging GmbH, Jena, Germany) was employed to observe their surface morphologies and measure the surface roughness (Ra). The qualification criteria for the machined workpieces were a smooth ground surface and a surface roughness of Ra ≤ 1.6 μm.

## 3. Results and Discussion

### 3.1. Brazed Diamond Micro-Powder Joint Morphology

**Figure 5:**
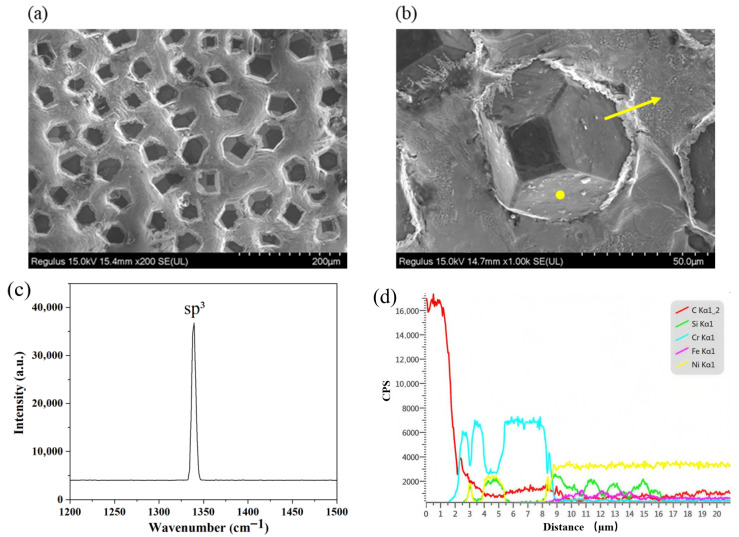
Brazed diamond micro-powder joint. (**a**) Brazed sample morphology, (**b**) brazed diamond micro-powder joint morphology, (**c**) Raman spectrum of the exposed surface of brazed diamond micro-powder, (**d**) the result of EDS line scan.

The morphology of the brazed diamond micro-powder joints is shown in Figure 5. As observed, the filler alloy was fully melted, and the surface of the brazed layer was free of cracks and pores. The diamond micro-powder particles were uniformly distributed in the filler layer with an appropriate exposure height, without agglomeration. Additionally, no fracture or erosion pits were observed on the exposed parts of the diamond micro-powder. Raman spectroscopy was employed to characterize the degree of graphitization on the exposed surfaces of the brazed diamond micro-powder (the position of the Raman test point is the yellow dot shown in Figure 5b), and the test results are presented in Figure 5c. Only a sharp characteristic peak of diamond at 1332 cm^−1^ was detected in the spectrum, which corresponds to the sp^3^ lattice structure of diamond. This indicates that no graphitization occurred on the exposed surfaces of the diamond micro-powder brazed with Ni-based filler alloy under the brazing process conditions adopted in this study. To investigate the elemental distribution characteristics at the bonding interface between diamond micro-powder and the filler layer, EDS line scan analysis was performed along the direction of the yellow arrow in Figure 5b, with the results presented in Figure 5d. The scanning range of 0~1.5 μm corresponds to diamond grains, dominated by C element. Within the 1.5~8 μm bonding interface region, the C content decreases significantly while the Cr content increases rapidly, indicating obvious segregation of Cr (from the brazing filler metal) on the diamond surface. Meanwhile, as the Cr content decreases in this interval, the contents of Ni and Si increase correspondingly, exhibiting a complementary distribution characteristic. Beyond 8 μm, the Cr content drops sharply, and Ni, Si, and Fe become the dominant elements, corresponding to the filler layer.

### 3.2. Microstructure at the Diamond Micro-Powder/Filler Alloy Interface

**Figure 6:**
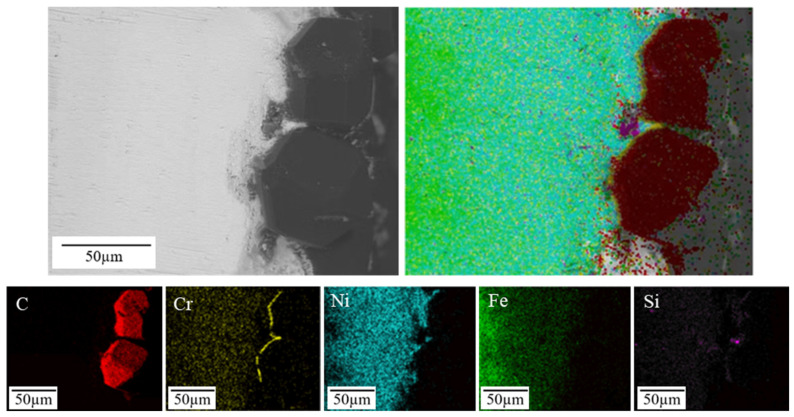
Internal microstructural morphology and elemental distribution of the brazed diamond micro-powder joint.

To investigate the elemental distribution characteristics inside the brazed diamond micro-powder joints, the cross-section of the joint was polished to prepare characterization samples. Figure 6 shows the internal microstructural morphology of the brazed diamond joint under the backscattered electron mode of SEM. From the figure, it can be observed that the internal structure of the joint was uniform and dense, with the brazed layer exhibiting excellent wettability to the diamond particles. According to the interfacial elemental distribution map, Cr element was highly enriched around the diamond particles, forming a continuous Cr-enriched region. Cr is a carbide-forming element. Previous studies have demonstrated that Cr can react with carbon on the diamond surface to form Cr–C compounds [29,30]. Consequently, Cr continuously segregates toward the diamond surface during brazing, resulting in the formation of a Cr-rich region near the diamond. Ni element was uniformly distributed within the brazed layer, while Si element did not exhibit obvious enrichment toward the diamond surface but rather formed Si-enriched regions inside the filler layer. Notably, the Ni-Si-enriched regions showed no spatial overlap with the continuous Cr-enriched region. None of the Fe-enriched regions were detected within the brazed layer, and the Fe concentration gradually increased with increasing proximity to the steel substrate. Thus, it can be concluded that the bonding interface between diamond micro-powder particles and the filler alloy consists of a continuous Cr-C compound interfacial layer and a Ni-Si-enriched brazed seam layer. Nevertheless, in-depth discussions regarding diffusion mechanisms, diffusivity of individual elements in the filler metal, and evolution of eutectic structures are not included in the present work, and these topics will be further explored in future research.

### 3.3. Microstructure at the Filler Alloy/Steel Substrate Interface

**Figure 7:**
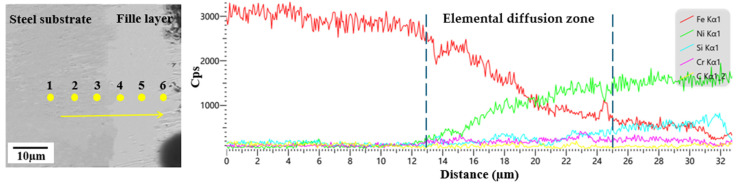
Morphology and EDS line scan of the bonding region between the steel substrate and Ni-based filler alloy.

Figure 7 presents the microstructural morphology and EDS line scan results of the bonding region between the steel substrate and the brazed layer. From the figure, it can be observed that the substrate and the filler layer were closely bonded, forming a distinct bonding transition zone. At the bonding interface, Cr, Si, and C elements exhibited no obvious diffusion or segregation, whereas Fe and Ni elements showed a significant concentration gradient, indicating intense elemental diffusion of the two elements near the interface. As the diffusion distance from the interface increased, the Fe concentration decreased rapidly, whereas Ni had a wider diffusion range with a relatively gentle concentration change. This difference stems from the fact that the steel substrate is in a solid state during brazing, which resulted in a low diffusion rate and narrow diffusion range of Fe atoms within it; in contrast, the brazing filler metal was in a molten state, which facilitated the diffusion and migration of Ni atoms. The EDS point scanning results are presented in Table 3. The variation trends of Fe, Ni, and Si at each test position are consistent with those obtained from the line scan. As the measurement points gradually transition from the substrate to the brazing layer, the Fe content decreases gradually, while the contents of Ni and Si increase correspondingly. The C content fluctuates among different points, which is mainly attributed to the presence of trace C in both the substrate and the brazing filler metal. Meanwhile, diamond micro-powder continuously releases dissolved carbon atoms into the filler alloy during the brazing process. Consequently, the C content in the bonding region fluctuates with the measurement position but shows no obvious systematic trend. Cr exists at a relatively low concentration in the steel substrate but is abundant in the brazing layer. Thus, the Cr content only fluctuates slightly at measurement point 3, while maintaining an increasing trend at other points as the measurement position approaches the brazing layer. Based on the elemental composition of test point 3, the bonding interface was dominated by Fe and Ni elements. Based on the analysis of the relevant literature [31], both Ni and Fe have a face-centered cubic (FCC) structure, with extremely close atomic radius and lattice constants, satisfying the conditions for mutual dissolution to form a solid solution. During brazing, a Ni-Fe substitutional solid solution was formed at the bonding interface between the Ni-based filler alloy and the steel substrate, thereby achieving chemical metallurgical bonding between them.

### 3.4. Interfacial Phase on Brazed Diamond Grits

**Figure 8:**
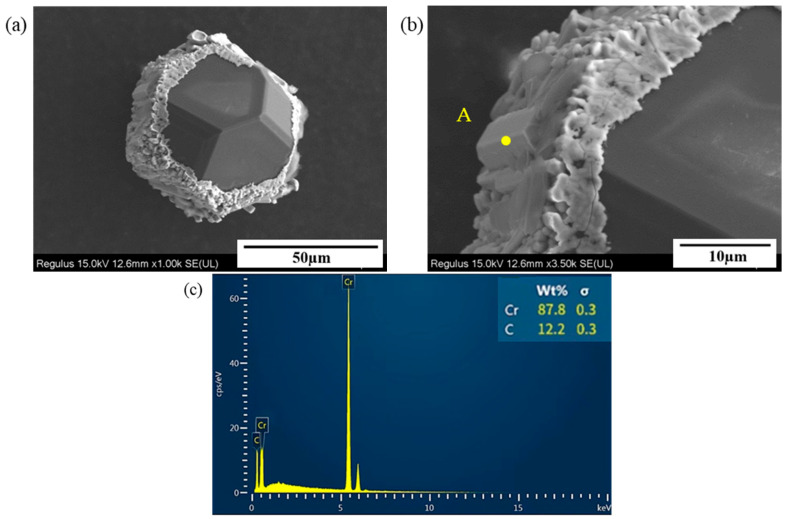
Interface products on brazed diamond micro-powder. (**a**) Overall morphology; (**b**) morphology of the compounds; (**c**) EDS analysis results of the interface products.

To identify the phase of Cr-C compounds formed around diamond particles, the brazed specimens were subjected to strong acid etching. Since both diamond and Cr-C compounds are insoluble in strong acid, the brazing filler metal layer could be completely removed while the diamond particles and the surface-formed compounds remained well preserved. Figure 8 shows the morphology of diamond micro-powders and reaction products. It can be observed that the brazed diamond micro-powders maintain intact crystal forms without obvious thermal erosion damage on the surface. A large number of lath-like compounds (designated as Phase A) are formed and attached to the diamond surface. Combined with EDS point scan results in which only Cr and C elements were detected in this compound, and by matching the atomic ratio of the two elements as well as the lath-like morphology, it is inferred that the lath-like compound was Cr_3_C_2_.

**Figure 9:**
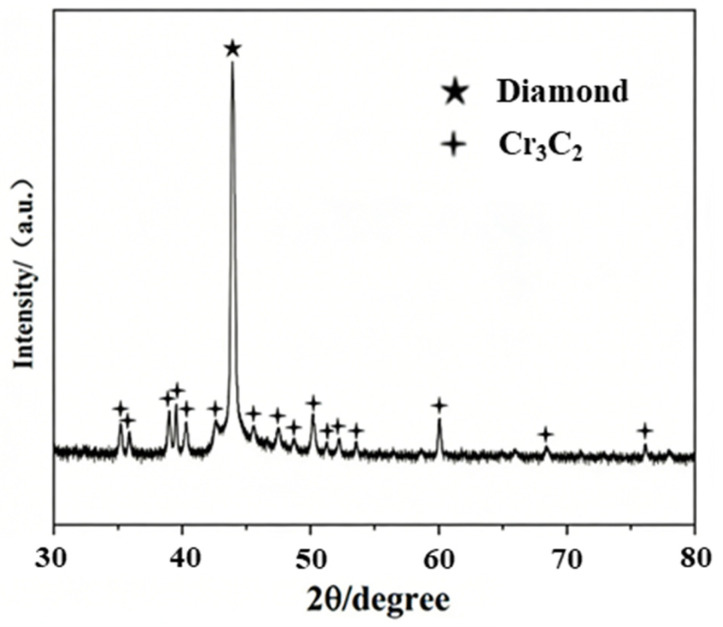
XRD analysis results of the brazed diamond.

To determine the phase of the reaction product, X-ray diffraction (XRD) analysis was performed on the isolated diamond particles after brazing, and the results are shown in Figure 9. XRD patterns indicate that apart from the characteristic strong diffraction peaks of diamond, only the diffraction peaks corresponding to Cr_3_C_2_ are observed. This confirms that only a single phase of lath-like Cr_3_C_2_ compound is formed on the diamond surface during the brazing process.

Thermodynamic calculations reveal that the Gibbs free energy for Cr_3_C_2_ formation is negative at the brazing temperature of 1025 °C, indicating that Cr_3_C_2_ can theoretically form spontaneously via reactions [32]. During brazing, C atoms on the surface of diamond micro-powders continuously dissolved and diffused into the filler alloy, interacted with Cr atoms through mutual attraction and reaction, and ultimately formed lath-like Cr_3_C_2_ on the diamond surface. The Cr_3_C_2_ layer effectively improves the wettability of Ni-based filler alloy to diamond. Additionally, Cr_3_C_2_ exhibits mixed bonding characteristics (covalent, ionic, and metallic bonds), which significantly mitigate differences in chemical properties and atomic bonding between diamond and Ni-based filler alloy, thereby reducing residual stress generation. Observations demonstrated that the Cr_3_C_2_ layer had a reasonable size and thickness, with its crystal morphology well matching the unit cell structure. Meanwhile, the filler layer climbed to a moderate height along the Cr_3_C_2_ on the diamond surface. This fully confirmed an appropriate reaction degree between the Ni-based filler alloy and diamond micro-powders, whereby excessive consumption of diamond micro-powder particles was avoided and the integrity and mechanical properties of the particles were well preserved.

### 3.5. Processing Performance of Brazed Diamond Micro-Powder Grinding Wheels

**Figure 10:**
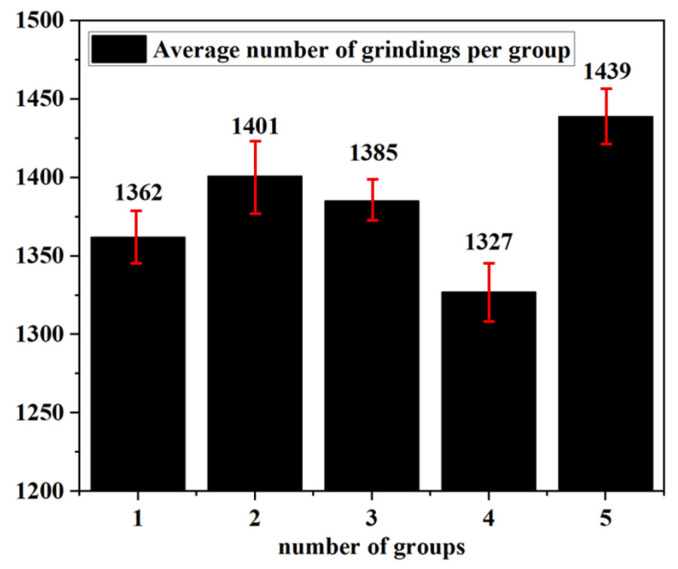
Service life of brazed diamond micro-powder grinding wheel.

Figure 10 presents the statistical service life of brazed diamond micro-powder grinding wheels for machining YG-6 cemented carbide. Under identical machining conditions, the number of YG-6 cemented carbide rods machined by 15 brazed diamond micro-powder grinding wheels in five processing groups before failure all exceeded 1300. Meanwhile, the standard deviation of each group was small and similar. Therefore, the as-prepared Ni-based brazed diamond micro-powder grinding wheel exhibits excellent service life and can meet the long-term machining requirements of YG-6 cemented carbide rods.

**Figure 11:**
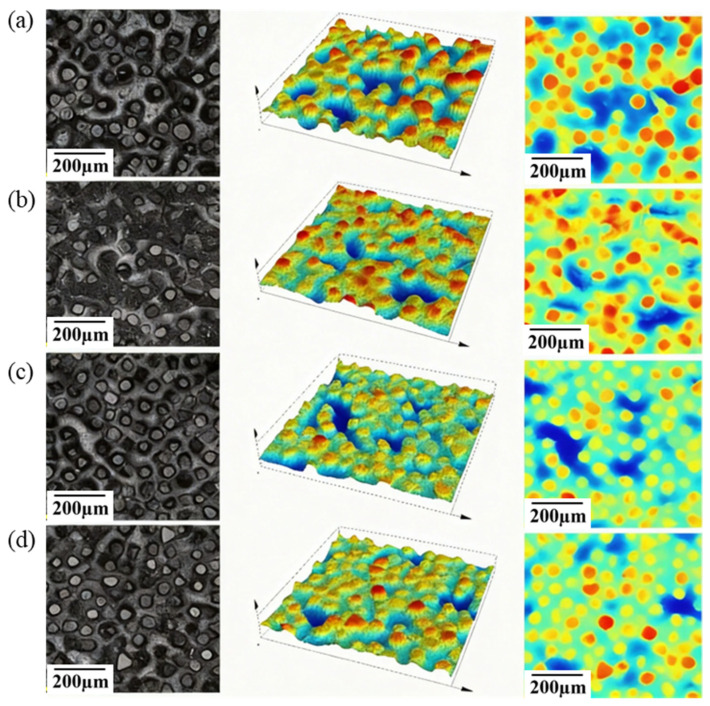
Morphology of brazed diamond micro-powder grinding wheel. (**a**) Original morphology of the end face; (**b**) post-grinding morphology of the end face; (**c**) original morphology of the lateral face; (**d**) post-grinding morphology of the lateral face.

To investigate the evolution of the surface morphology of brazed diamond micro-powder grinding wheels during the grinding process, morphological characterization was randomly performed on the working regions of the end face and lateral face of the grinding wheel after 500 grinding cycles. The surface morphology of the grinding wheel following 500 grinding passes is presented in Figure 11. As observed from the figure, the diamond protrusion height on both the end face and lateral face of the grinding wheel decreased to a certain extent, whereas the diamond micro-powder particles remained tightly bonded to the brazing filler layer with no occurrence of premature particle detachment. Meanwhile, cleavage fracture of diamond micro-powder particles along the bonding interface was also rarely detected. These results demonstrate that an excellent bonding strength is achieved among the diamond micro-powder, brazing filler layer and steel substrate, and the diamond micro-powder suffers a low degree of thermal damage during the brazing process. The end face of the grinding wheel was subjected to a greater grinding impact force. Additionally, the stress direction of diamond micro-powder particles on the end face altered as inclined steps were formed during grinding, which further exacerbated the wear of these particles and ultimately resulted in a higher wear degree of diamond micro-powder on the end face relative to the lateral face.

The average surface roughness of the as-processed YG-6 cemented carbide rods (No. 250#, 500#, 750#, 1000#, and 1250#) was measured, and its values were compared with those of the pristine rods; the detailed measurement results are presented in Table 4. As shown in Table 4, the surface roughness of all processed rods was significantly higher than that of the pristine counterparts, which is mainly attributed to the non-uniform distribution of diamond microparticles on the tool surface and the inconsistent protrusion heights of diamond grains. Furthermore, the random participation of diamond microparticles in the grinding process contributed to a further increase in surface roughness. Nevertheless, the average surface roughness (Ra) of workpieces at different processing stages remains below 1.6 μm with a small standard deviation. Accordingly, the brazed diamond micro-powder grinding wheel fabricated in this study meets the surface roughness requirements for the grinding of YG-6 cemented carbide. During the grinding process, with the continuous progression of grinding, the surface roughness of the cemented carbide rods exhibits a trend of first decreasing and then increasing. This phenomenon is determined by the wear characteristics of the brazed diamond grinding wheel during grinding. At the initial stage of grinding, the disparate protrusion heights of diamond grains resulted in a small number of effectively engaged diamond particles, leading to a relatively high surface roughness of the rods. As grinding proceeded, the number of diamond particles involved in material removal gradually increased, and the grinding process entered a stable stage. In this stage, diamond wear is dominated by microfracture, which gradually reduces the surface roughness of the rods. When the grinding process entered the late stage of intensified wear, diamond grains underwent significant wear aggravation; meanwhile, grain passivation reduced the number of effectively engaged diamond particles for grinding, which in turn caused the surface roughness of the rods to increase until the grinding wheel failed.

**Figure 12:**
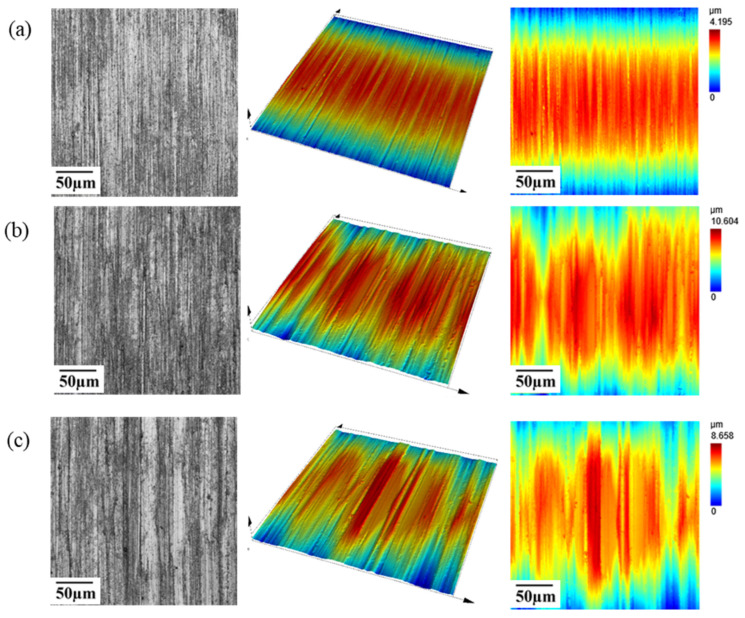
Surface monography of the workpiece. (**a**) Original surface topography; (**b**) end grinding surface monography; (**c**) step grinding surface monography.

Figure 12 shows the surface morphologies of the 500# rod before and after processing. As can be observed from the figure, compared with the pristine surface, the diamond microparticles on the grinding wheel exert sliding, plowing, and cutting effects on the workpiece surface, resulting in grinding marks with an approximately strip-like texture structure, accompanied by surface undulation characteristics on the texture. Due to the randomness of the protrusion height of the distributed abrasive grains, the formed strip-like grinding textures exhibit obvious peak–valley characteristics and uneven scratch depths, where the scratch depth in some areas is significantly greater than that in the surrounding areas. Furthermore, the coolant has excellent lubricating and flushing effects. It can not only effectively reduce the friction force and frictional heat between the grinding wheel and the workpiece, slowing down the wear rate of the grinding wheel, but can also carry the fractured and detached abrasive grains and grinding chips to flow out. This reduces the interference of impurities on the grinding process and thereby effectively decreases the surface roughness of the workpiece.

## 4. Conclusions

In this study, a brazed diamond micro-powder grinding wheel was successfully fabricated using Ni-based filler metal. The interfacial microstructure characteristics of the brazed joints and the machining performance of the grinding wheel were systematically investigated. The as-prepared grinding wheel realized one-step precision grinding of YG-6 WC-Co cemented carbide. The main conclusions are drawn as follows:

(1) The brazed diamond micro-powder is uniformly distributed on the steel substrate with a moderate protrusion height. Meanwhile, no graphitization occurs on the exposed surface of the brazed diamond, and the diamond exhibits no severe thermal damage during the brazing process. This fully demonstrates that the brazing process adopted in this study is reasonable and reliable, providing important technical support for the subsequent fabrication of other types of brazed diamond micro-powder tools.

(2) The active element Cr in the filler metal reacts with C atoms on the surface of diamond micro-powder at the bonding interface, forming a continuous Cr_3_C_2_ compound layer. This Cr_3_C_2_ layer improves the wettability between the Ni-based alloy and diamond, and realizes the chemical metallurgical bonding between diamond and the Ni-based brazing layer. This also guarantees the excellent service life of the brazed micro-powder diamond grinding wheel during the grinding of cemented carbide.

(3) A dense metallurgical bond was formed between the Ni-based alloy and the steel substrate via elemental diffusion. Obvious concentration gradients of Fe and Ni exist at the interface, and a Ni–Fe substitutional solid solution was formed. The well-defined interfacial transition zone ensures a reliable connection between the brazing layer and the steel substrate.

(4) The brazed diamond micro-powder grinding wheel exhibits excellent service life and bonding reliability, and a single wheel can stably machine more than 1300 YG-6 cemented carbide rods. During grinding, diamond particles are firmly bonded to the brazing layer without premature pull-out or interfacial brittle fracture. This wheel enables one-step precision grinding of YG-6 cemented carbide, and the surface roughness of the workpiece is always below 1.6 μm, meeting the requirements of precision machining.

## Figures and Tables

**Table 1:** Chemical composition of the 45# steel substrate (wt.%).

Type	C	Si	Cr	P	Mn	Ni	S	Fe
45#	0.42–0.50	≤0.35	≤0.25	≤0.035	0.50–0.80	≤0.25	≤0.35	Bal

**Table 2:** Mechanical properties of YG-6 cemented carbide.

Properties	WC-6Co
Density (g/cm^3^)	14.9–15.0
Hardness (HV)	1400–1600
Compressive strength (MPa)	4250
Transverse fracture strength (MPa)	1650–3000
Elastic modulus (GPa)	620

**Table 3:** EDS point analysis results at various positions in Figure 5 (wt.%).

Position	Fe	Ni	Cr	Si	C
1	97.35	1.55	0.06	0.63	0.41
2	96.93	2.32	0.20	0.17	0.38
3	66.39	30.77	2.04	0.39	0.41
4	53.06	43.31	1.63	1.56	0.44
5	30.13	64.62	3.03	1.86	0.36
6	18.29	74.06	3.14	4.09	0.42

**Table 4:** Surface roughness of the processed workpiece.

Processing Sequence Number	Unprocessed	250#	500#	750#	1000#	1250#	Standard Deviation
Average roughness of the end grinding surface (μm)	0.708	1.372	1.334	1.328	1.339	1.345	0.0171
Average surface roughness of the step grinding surface (μm)	-	1.157	1.125	1.116	1.132	1.148	0.0167

## Data Availability

The original contributions presented in this study are included in the article. Further inquiries can be directed to the corresponding author.
